# Supplementary material for: From sequence to enzyme mechanism using multi-label machine learning
Source: BMC Bioinformatics. 2014 May 19;15:150. doi: 10.1186/1471-2105-15-150 (PMC4229970; doi:10.1186/1471-2105-15-150)
Supplement: Additional file 2 — Java code of ml2db. Additional file ml2db_code.tar.gz contains the Java source code to run the multi-label machine learning experiments and save the results to database. The code’s Javadoc is included. [file 1471-2105-15-150-S2.zip › additional file 2/ml2db/ml2db/doc/main/ml2db/mulan/learn/MulanLearners.html]

MulanLearners


JavaScript is disabled on your browser.


- Overview
- Package
- Class
- Use
- Tree
- Deprecated
- Index
- Help

- Prev Class
- Next Class

- Frames
- No Frames

- All Classes

- Summary:
- Nested |
- Field |
- Constr |
- Method

- Detail:
- Field |
- Constr |
- Method


main.ml2db.mulan.learn

## Class MulanLearners

- java.lang.Object
- - main.ml2db.mulan.learn.MulanLearners

- ---

    

  ```
  public class MulanLearners
  extends java.lang.Object
  ```

  Class

  Version:
  :   16 Jun 2010

  Author:
  :   Luna De Ferrari ldf(at)st-andrews.ac.uk

- - ### Field Summary

    Fields

    | Modifier and Type | Field and Description |
    | --- | --- |
    | `static int` | `K1` |
    | `static int` | `K5` |
    | `static boolean` | `m_debug` |
    | `static double` | `N1` |
  - ### Constructor Summary

    Constructors

    Constructor and Description

    `MulanLearners()`
  - ### Method Summary

    Methods

    | Modifier and Type | Method and Description |
    | --- | --- |
    | `static MulanLearner` | `getBinaryRelevanceWrappedClassifier(weka.classifiers.Classifier classifier, java.lang.String classifierName)` |
    | `static MulanLearner` | `getBPMLLBackPropagation()` |
    | `static MulanLearner` | `getBRk1NN()` (Binary Relevance Nearest Neighbours) |
    | `static MulanLearner` | `getBRk1NNAL()` (Binary Relevance Nearest Neighbours) |
    | `static MulanLearner` | `getBRk1NNALJaccard()` (Binary Relevance Nearest Neighbours) |
    | `static MulanLearner` | `getBRk1NNALJaccardEuclidean()` Binary Relevance Nearest Neighbours with jaccard+euclidean distance |
    | `static MulanLearner` | `getBRk1NNManhattan()` Binary Relevance Nearest Neighbours learner (Active Learning version) with Manhattan distance instead of default Euclidean distance. |
    | `static MulanLearner` | `getBRk2NNManhattan()` |
    | `static MulanLearner` | `getBRk5NNAL()` (Binary Relevance Nearest Neighbours) |
    | `static MulanLearner` | `getBRkNN(int neighbours)` public static MulanLearner getBRk1NNAL() { return MulanLearner.getBRkNNAL(MulanLearners.K1); } |
    | `static MulanLearner` | `getBRkNNAL(int neighbours)` |
    | `static MulanLearner` | `getEnsembleOfClassifierChains(int numberOfModels)` The Classifier Chain model ( CC ) involves \| L \| binary classifiers as in BM . |
    | `static MulanLearner` | `getHierarchicalMultilabel(mulan.classifier.MultiLabelLearnerBase multiLabelClassifier, java.lang.String classifierName)` |
    | `static MulanLearner` | `getHomer()` |
    | `static MulanLearner` | `getLabelPowersetJ48C45()` From Wikipedia: C4.5 is an algorithm used to generate a decision tree developed by Ross Quinlan. |
    | `static MulanLearner` | `getLabelPowersetSMO()` |
    | `static MulanLearner` | `getLabelPowersetSMOReg()` |
    | `static MulanLearner` | `getLabelPowersetWrappedClassifier(weka.classifiers.Classifier classifier, java.lang.String classifierName)` |
    | `static java.util.Vector<MulanLearner>` | `getLearners()` |
    | `static MulanLearner` | `getNaiveBayesBinaryRelevance()` |
    | `static MulanLearner` | `getNaiveBayesLabelPowerset()` |
    | `static MulanLearner` | `getOneRule()` |
    | `static MulanLearner` | `getPerceptron()` |
    | `static MulanLearner` | `getRakel_J48_C45()` From Wikipedia: C4.5 is an algorithm used to generate a decision tree developed by Ross Quinlan. |
    | `static MulanLearner` | `getRakelWrappedClassifier(mulan.classifier.MultiLabelLearner multiLabelClassifier, java.lang.String classifierName)` RAKEL-MLKNN (RAndom k-labELsets) // failed not enough labels in metadata |
    | `static MulanLearner` | `getZeroRule()` public static MulanLearner getWrappedClassifier(TransformationBasedMultiLabelLearner wrapper, String wrapperName, Classifier classifier, String classifierName){ TransformationBasedMultiLabelLearner wrapped = new TransformationBasedMultiLabelLearner(classifier); wrapper.setDebug(true); return new MulanLearner(MulanLearner.getName(wrapper), wrapperName + "\_" + classifierName, wrapped); } |
    | `static void` | `main(java.lang.String[] args)` |

    - ### Methods inherited from class java.lang.Object

      `equals, getClass, hashCode, notify, notifyAll, toString, wait, wait, wait`

- - ### Field Detail


    - #### m\_debug

      ```
      public static boolean m_debug
      ```


    - #### K1

      ```
      public static int K1
      ```


    - #### K5

      ```
      public static int K5
      ```


    - #### N1

      ```
      public static double N1
      ```
  - ### Constructor Detail


    - #### MulanLearners

      ```
      public MulanLearners()
      ```
  - ### Method Detail


    - #### getBinaryRelevanceWrappedClassifier

      ```
      public static MulanLearner getBinaryRelevanceWrappedClassifier(weka.classifiers.Classifier classifier,
                                                     java.lang.String classifierName)
      ```


    - #### getBPMLLBackPropagation

      ```
      public static MulanLearner getBPMLLBackPropagation()
      ```


    - #### getBRk1NN

      ```
      public static MulanLearner getBRk1NN()
      ```

      (Binary Relevance Nearest Neighbours)

      Returns:


    - #### getBRk1NNAL

      ```
      public static MulanLearner getBRk1NNAL()
      ```

      (Binary Relevance Nearest Neighbours)

      Returns:


    - #### getBRk1NNALJaccard

      ```
      public static MulanLearner getBRk1NNALJaccard()
      ```

      (Binary Relevance Nearest Neighbours)

      Returns:


    - #### getBRk1NNALJaccardEuclidean

      ```
      public static MulanLearner getBRk1NNALJaccardEuclidean()
      ```

      Binary Relevance Nearest Neighbours with jaccard+euclidean distance

      Returns:


    - #### getBRk1NNManhattan

      ```
      public static MulanLearner getBRk1NNManhattan()
      ```

      Binary Relevance Nearest Neighbours learner (Active Learning version)
      with Manhattan distance instead of default Euclidean distance.

      Returns:


    - #### getBRk2NNManhattan

      ```
      public static MulanLearner getBRk2NNManhattan()
      ```


    - #### getBRk5NNAL

      ```
      public static MulanLearner getBRk5NNAL()
      ```

      (Binary Relevance Nearest Neighbours)

      Returns:


    - #### getBRkNN

      ```
      public static MulanLearner getBRkNN(int neighbours)
      ```

      public static MulanLearner getBRk1NNAL() { return
      MulanLearner.getBRkNNAL(MulanLearners.K1); }


    - #### getBRkNNAL

      ```
      public static MulanLearner getBRkNNAL(int neighbours)
      ```


    - #### getEnsembleOfClassifierChains

      ```
      public static MulanLearner getEnsembleOfClassifierChains(int numberOfModels)
      ```

      The Classifier Chain model ( CC ) involves | L | binary classifiers as in
      BM . Classifiers are linked along a chain where each classifier deals
      with the binary relevance problem associated with label l j ∈ L .

      Parameters:
      :   `numberOfModels` -

      Returns:


    - #### getHierarchicalMultilabel

      ```
      public static MulanLearner getHierarchicalMultilabel(mulan.classifier.MultiLabelLearnerBase multiLabelClassifier,
                                           java.lang.String classifierName)
      ```


    - #### getHomer

      ```
      public static MulanLearner getHomer()
      ```


    - #### getLabelPowersetJ48C45

      ```
      public static MulanLearner getLabelPowersetJ48C45()
      ```

      From Wikipedia: C4.5 is an algorithm used to generate a decision tree
      developed by Ross Quinlan. C4.5 is an extension of Quinlan's earlier ID3
      algorithm.

      Returns:


    - #### getLabelPowersetSMO

      ```
      public static MulanLearner getLabelPowersetSMO()
      ```


    - #### getLabelPowersetSMOReg

      ```
      public static MulanLearner getLabelPowersetSMOReg()
      ```


    - #### getLabelPowersetWrappedClassifier

      ```
      public static MulanLearner getLabelPowersetWrappedClassifier(weka.classifiers.Classifier classifier,
                                                   java.lang.String classifierName)
      ```


    - #### getLearners

      ```
      public static java.util.Vector<MulanLearner> getLearners()
      ```

      Returns:


    - #### getNaiveBayesBinaryRelevance

      ```
      public static MulanLearner getNaiveBayesBinaryRelevance()
      ```


    - #### getNaiveBayesLabelPowerset

      ```
      public static MulanLearner getNaiveBayesLabelPowerset()
      ```


    - #### getOneRule

      ```
      public static MulanLearner getOneRule()
      ```


    - #### getPerceptron

      ```
      public static MulanLearner getPerceptron()
      ```


    - #### getRakel\_J48\_C45

      ```
      public static MulanLearner getRakel_J48_C45()
      ```

      From Wikipedia: C4.5 is an algorithm used to generate a decision tree
      developed by Ross Quinlan. C4.5 is an extension of Quinlan's earlier ID3
      algorithm.

      Returns:


    - #### getRakelWrappedClassifier

      ```
      public static MulanLearner getRakelWrappedClassifier(mulan.classifier.MultiLabelLearner multiLabelClassifier,
                                           java.lang.String classifierName)
      ```

      RAKEL-MLKNN (RAndom k-labELsets) // failed not enough labels in metadata

      Parameters:
      :   `multiLabelClassifier` -

      Returns:


    - #### getZeroRule

      ```
      public static MulanLearner getZeroRule()
      ```

      public static MulanLearner
      getWrappedClassifier(TransformationBasedMultiLabelLearner wrapper, String
      wrapperName, Classifier classifier, String classifierName){
      TransformationBasedMultiLabelLearner wrapped = new
      TransformationBasedMultiLabelLearner(classifier); wrapper.setDebug(true);
      return new MulanLearner(MulanLearner.getName(wrapper), wrapperName + "\_"
      + classifierName, wrapped); }


    - #### main

      ```
      public static void main(java.lang.String[] args)
      ```


- Overview
- Package
- Class
- Use
- Tree
- Deprecated
- Index
- Help

- Prev Class
- Next Class

- Frames
- No Frames

- All Classes

- Summary:
- Nested |
- Field |
- Constr |
- Method

- Detail:
- Field |
- Constr |
- Method
